# Comparison of separation methods for tissue‐derived extracellular vesicles in the liver, heart, and skeletal muscle

**DOI:** 10.1002/2211-5463.13075

**Published:** 2021-01-29

**Authors:** Adam Matejovič, Shohei Wakao, Masaaki Kitada, Yoshihiro Kushida, Mari Dezawa

**Affiliations:** ^1^ Department of Stem Cell Biology and Histology Tohoku University Graduate School of Medicine Sendai Japan; ^2^ Department of Virology Tohoku University Graduate School of Medicine Sendai Japan; ^3^ Department of Anatomy Kansai Medical University Osaka Japan

**Keywords:** exosome, extracellular vesicles, heart, liver, muscle, tissue, ultracentrifugation

## Abstract

Extracellular vesicles (EVs), which are nanosized vesicles released by cells as intracellular messengers, have high potential as biomarkers. EVs are usually collected from *in vitro* sources, such as cell culture media or biofluids, and not from tissues. Techniques enabling direct collection of EVs from tissues will extend the applications of EVs. We compared methods for separating EVs from solid liver, heart, and skeletal muscle. Compared with a precipitation method, an ultracentrifugation‐based method for collection of EVs from solid tissues yielded a higher proportion of EVs positive for EV‐related markers, with minimum levels of intracellular organelle‐related markers. Some tissue‐specific modifications, such as a sucrose cushion step, may improve the yield and purity of the collected EVs.

## Graphical abstract

Extracellular vesicle (EV)‐related research is mostly focused on cell culture and biofluids. Recently published studies reported the collection of EVs from a single tissue type. We compared several separation methods and collected vesicles from three different types of solid tissue—liver, skeletal muscle, and heart. EV characterization followed the criteria of the International Society of Extracellular Vesicles (ISEV).

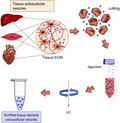

**Abbreviations**
Alix: programmed cell death 6‐interacting protein
EVs: extracellular vesicles
HSP70: heat‐shock 70 kDa protein
RPL5: ribosomal protein L5
TEM: transmission electron microscopy
TRPS: tunable resistive pulse sensing
TSG101: tumor susceptibility gene 101 protein

Extracellular vesicles (EVs), including exosomes and microvesicles, are small nanovesicles released from all cell types. Several subtypes of EVs exist, differing in their composition and biogenesis, and often overlapping in size. EVs are classified into three main categories: (a) exosomes with an endosomal origin (50–150 nm), (b) microvesicles formed by outward budding of the plasma membrane (50–1000 nm), and (c) apoptotic bodies (800–5000 nm) released from cells undergoing apoptosis [1, 2, 3].

Since the discovery of major roles of EVs, namely packing bioactive materials (including mRNA, microRNA, signaling molecules, and enzymes) and transferring these materials to recipient cells to mediate intercellular communications [4], they have attracted high attention across many fields. Their potential as promising disease biomarkers relies on the characteristics of the cells that produce them, with the content changing under pathological or physiological conditions [5]. EVs are abundantly released following tissue damage [6, 7] and affect tissue stem cell activity, disease remodeling, and tissue regeneration [8, 9]. Most studies to date have evaluated EVs collected from *in vitro* sources, such as cell culture media or biofluids, and not tissues. Techniques enabling direct collection of EVs from tissues will be important for future applications as the study of tissue EVs is on the increase [10].

While polymer‐based precipitation kits are easy‐to‐use tools for separating the components of cell culture media and biofluids, ultracentrifugation remains the standard approach [9]. The multicellular nature of tissues produces different physical and chemical properties, and may require different technical concepts, particularly for tissue processing prior to EV collection. Several different methods were reported for the thymus and spleen [11], brain [12, 13, 14], muscle [15], apex of the heart [7], and adipose tissue [6]. The methods used in these studies were based on either (a) tissue mincing and/or enzymatic homogenization, or (b) combining differential centrifugation with other methods—triple sucrose cushion [13], sucrose gradient [12], repelleting [15], repelleting and sucrose gradient [6], or precipitation [7]. Additionally, the methods varied with respect to the applied centrifugation force (25 000 to 270 000 ***g***) and time (45 min–16 h), repetitions (1–3×), and tissue types. Methods based on sucrose gradients tend to be labor‐intense, whereas precipitation kits are easy‐to‐use.

In the present study, we investigated various approaches for collecting EVs from three different solid tissues—liver, skeletal muscle, and heart.

## Materials and methods

### Animal damage models

C57BL/6N mice (male, 10–12 weeks old, 21–26 g) were used for all the tissue damage models (three animals for acute liver damage, three for skeletal muscle degeneration, and 22 for acute cardiomyopathy). All animals were treated according to the regulations of the Standards for Humane Care and Use of Laboratory Animals of Tohoku University. The animal experiments were approved by the Animal Care and Experimentation Committee of Tohoku University, Graduate School of Medicine (Permission No. 103‐2).

Because tissue damage is reported to trigger an abundant release of EVs compared with intact tissue [6, 7], we induced tissue damage in the liver, muscle, and heart as described below.

#### Acute liver damage model

Damage was induced by a single intraperitoneal injection of carbon tetrachloride (CCL_4_; 2 µL·g^−1^ body weight) dissolved in olive oil (1 : 10 v/v), according to a previous report [16], and the liver was collected 24 h after injection.

#### Skeletal muscle degeneration model

Under deep anesthesia (intraperitoneal injection of medetomidine 0.3 mg·kg^−1^, midazolam 4 mg·kg^−1^, and butorphanol tartrate 5 mg·kg^−1^), cardiotoxin (10 μm diluted in PBS) was injected into the thigh and calf muscles at multiple locations (100 µL per injection in three different areas). Muscles were collected 24 h after the injections.

#### Acute cardiomyopathy model

Damage was induced by a single intraperitoneal injection of doxorubicin hydrochloride (20 mg·kg^−1^; #04021521; Fujifilm Wako, Tokyo, Japan) resuspended in saline, as described previously [17]. The whole heart was collected 5 days after the injection.

### Collection of EVs from damaged liver

**Fig. 1:**
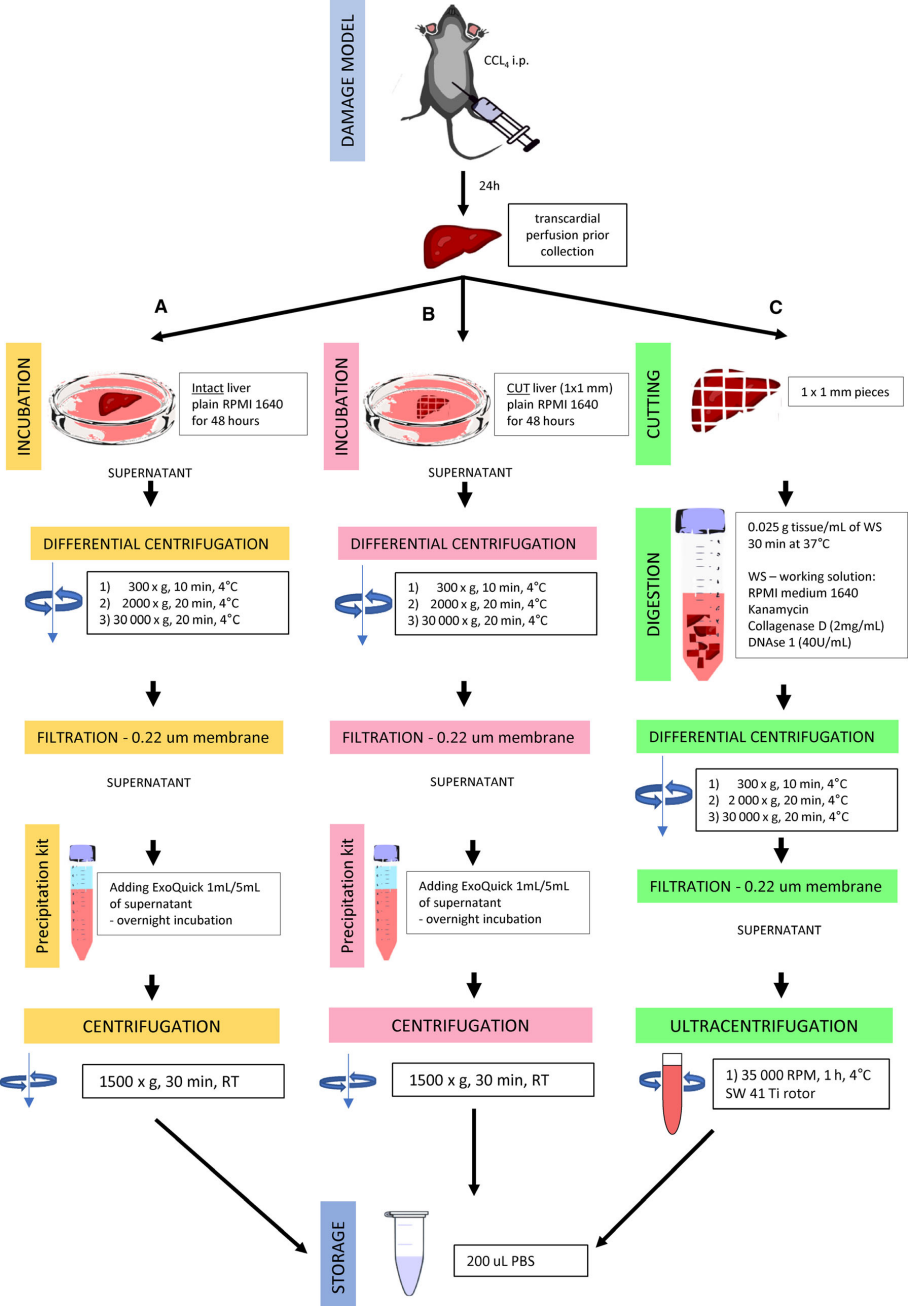
Schematic of 3 EV collection protocols for damaged liver tissue. Mice received a single intraperitoneal injection of carbon tetrachloride (CCL_4_; 2 µL·g^−1^ of body weight). After 24 h, animals were transcardially perfused with 20 mL of 1× PBS under deep anesthesia and the liver was dissected out. The liver was subjected to one of the three protocols; (A) Protocol A (ExoQuick + intact liver), (B) Protocol B (ExoQuick + cut liver), and (C) Protocol C (ultracentrifugation).

To collect EVs from the damaged liver, we applied three different protocols as described below (Fig. 1). One whole liver was used for each protocol, and each protocol was repeated three times. Therefore, three livers were used for each protocol. We applied either a precipitation kit or ultracentrifugation to determine the better protocol for collecting EVs. Before tissue collection, each animal was transcardially perfused with 20 mL of 1× PBS under deep anesthesia (intraperitoneal injection of medetomidine 0.3 mg·kg^−1^, midazolam 4 mg·kg^−1^, and butorphanol tartrate 5 mg·kg^−1^). The liver was immediately stored in 20 mL RPMI 1640 medium (#11875093; Gibco, Thermo Scientific, Waltham, MA, USA), stored on ice, and processed according to the protocols shown in Fig. 1.

#### Protocol A

The whole liver (~ 0.970 g) with 20 mL RPMI 1640 medium was incubated in a 10‐cm dish at 37 °C in 5% CO_2_ for 48 h [18]. The medium (18 mL) was then collected and subjected to differential centrifugation: 300 ***g*** for 10 min, 2000 ***g*** for 20 min, and 30 000 ***g*** for 20 min, all at 4 °C (Kubota 7780, AG‐508CA Rotor, Osaka, Japan). The supernatants were collected by decanting. The supernatant was filtered through a 0.22‐μm membrane (Merck Millipore, Burlington, MA, USA) and incubated overnight with ExoQuick‐TC (#EXOTC10A‐1; System Biosciences, Palo Alto, CA, USA) at 4 °C (2 mL mixed with 10 mL of supernatant). The next day, the sample was centrifuged at 1500 ***g*** for 30 min (room temperature) and the pellet was resuspended in 200 µL of filtered 1× PBS. An aliquot (40 µL) was stored at −80 °C for later analysis by transmission electron microscopy (TEM). The remaining sample was immediately used for western blot and tunable resistive pulse sensing (TRPS) analysis.

#### Protocol B

The whole liver (~ 1.075 g) with 20 mL RPMI 1640 medium was transferred to a 10‐cm dish, dissected into ~ 1 × 1 mm pieces using a razor, and cultured as an explant for 48 h. The culture medium was then subjected to the same procedure as in Protocol A.

#### Protocol C

The sample was collected using a centrifugation‐based protocol as described by Jang *et al*. [19] without purification of the EV pellet by isopycnic centrifugation with an iodixanol gradient. Briefly, the whole liver (~ 1.04 g) in 20 mL RPMI 1640 medium was transferred to 10‐cm dish, cut into ~ 1 × 1 mm pieces, and mixed with working solution (1 mL working solution/0.025 g tissue)—RPMI 1640 medium containing 2 mg·mL^−1^ collagenase D (#11088858001; Roche, Basel, Switzerland) and 40 U·mL^−1^ DNase I (#11284932001; Roche). After 30 min of incubation at 37 °C, the sample (cut liver pieces in 40 mL working solution) was subjected to differential centrifugation: 300 ***g*** for 10 min, 2000 ***g*** for 20 min, and 30 000 ***g*** for 20 min, all at 4 °C (Kubota 7780, AG‐508CA Rotor), and filtered through a 0.22‐μm membrane. The supernatant was centrifuged at 35 000 r.p.m. (210 053 ***g***) for 1 h at 4 °C (SW41 Rotor, Optima LX‐80; Beckman Coulter, Brea, CA, USA). The pellet was resuspended in 200 µL filtered 1× PBS. An aliquot (40 µL) was stored at −80 °C for later analysis by TEM. The remaining sample was immediately used for western blot and TRPS analysis.

### Collection of damaged skeletal muscle‐derived EVs

Muscle tissue from both legs (~ 1.4 g) was dissected out after transcardial perfusion with 20 mL of 1× PBS under deep anesthesia (intraperitoneal injection of medetomidine 0.3 mg·kg^−1^, midazolam 4 mg·kg^−1^, and butorphanol tartrate 5 mg·kg^−1^) and immediately stored in 20 mL RPMI 1640 medium on ice until further processed according to Protocol C. This was repeated three times. The total amount of supernatant used for ultracentrifugation from a single collection was ~ 40 mL. The supernatant was discarded, and the pellet was resuspended in 200 μL of filtered 1× PBS and an aliquot (40 μL) was stored at −80 °C for TEM. The remaining sample was immediately used for western blot and TRPS.

### Collection of damaged heart‐derived EVs

Hearts were collected from mice under deep anesthesia (as described above), washed in 1× PBS to remove the blood, and immediately stored in RPMI 1640 medium on ice. A total of 22 hearts were processed using one of two protocols (11 hearts/protocol with a total weight of 1.45 and 1.39 g, respectively):

#### Protocol C method (ultracentrifugation‐based)

Hearts were enzymatically digested and processed following Protocol C. The obtained pellet was resuspended in 200 µL filtered 1× PBS, an aliquot (40 µL) was stored at −80 °C for TEM, and the remaining sample was analyzed by western blot and TRPS.

#### Protocol C + sucrose (combination of ultracentrifugation and sucrose cushion)

Hearts were processed as in Protocol C with one modification—after the differential centrifugation and filtration, the supernatant was loaded on a 1‐mL layer of 30% sucrose solution (called a sucrose cushion) according to Gupta *et al*. [20] (prepared in 1× PBS) and ultracentrifuged at 35 000 r.p.m. (210 053 ***g***) for 1 h at 4 °C (SW41 Rotor, Optima LX‐80; Beckman Coulter). The sucrose fraction containing the EVs was then resuspended in 1× PBS and washed by ultracentrifugation again at 35 000 r.p.m. for 1 h at 4 °C. The final pellet was resuspended in 200 µL of filtered 1× PBS, an aliquot (40 µL) was stored at −80 °C for TEM, and the remaining sample was analyzed by western blot and TRPS.

### Western blot

For protein quantification, samples were lysed with RIPA Lysis and Extraction Buffer (#89900,;Thermo Scientific) and measured using a Pierce bicinchoninic acid Protein Assay Kit (#23227; Thermo Scientific). Each sample was mixed with 2× reducing sample buffer (#30566‐22; Nacalai Tesque, Kyoto, Japan) and heated at 95 °C for 10 min. Equal amounts of samples and positive controls (14 or 7 μg for Protocols A, B, and C in the liver; 10 μg for the skeletal muscle; and 10 or 6 μg for the heart) were loaded on to 10% precast SDS/PAGE gels (#195‐14951; FUJIFILM Wako) under reducing conditions. Proteins were transferred to a polyvinylidene difluoride membrane (Merck Millipore) and blocked in 2.5% skim milk for 1 h before overnight incubation with the following antibodies: rabbit polyclonal anti‐CD63 (1 : 1000 dilution, #GTX17441; GeneTex, Irvine, CA, USA), rabbit monoclonal anti‐programmed cell death 6‐interacting protein (Alix; 1 : 2500 dilution, #ab186429; Abcam, Cambridge, UK), rabbit monoclonal anti‐heat‐shock 70 kDa protein (HSP70; 1 : 1000 dilution, #ab181606; Abcam), mouse monoclonal anti‐tumor susceptibility gene 101 protein (TSG101; 1 : 1000 dilution, #ab83; Abcam), rabbit polyclonal anti‐calnexin (1 : 10 000, #ab22595; Abcam), and rabbit polyclonal anti‐RPL‐5 (1 : 1000, #14568; Cell Signaling, Danvers, MA, USA), all in blocking solution at 4 °C under constant rocking. Membranes were washed three times with 1× Tris‐buffered saline Tween 20 (TBS‐T) and then incubated with mouse (#115‐035‐071; Jackson ImmunoResearch, West Grove, PA, USA) or rabbit (#111‐035‐144; Jackson ImmunoResearch) horseradish peroxidase‐conjugated secondary antibodies (1 : 5000 dilution) in blocking solution for 1 h at room temperature. After washing the membranes three times with TBS‐T (3× 10 min each), the blots were developed using an ECL detection system (#32132; Thermo Scientific) and visualized on an LAS‐4000 Imaging System (FUJIFILM). Positive controls were prepared as whole‐cell lysates from HeLa (ATCC® CCL‐2™) for TSG101, HSP70, Alix, calnexin, and ribosomal protein L5 (RPL5); and HEK293T (ATCC® CCL‐2™) for CD63, as described above. Proteins from whole‐cell lysates were extracted in RIPA Lysis and Extraction Buffer (#89900; Thermo Scientific) according to the manufacturer’s protocol.

### Tunable resistive pulse sensing

The concentration and size distribution of the obtained samples were determined by qNano (Izon Science, Christchurch, New Zealand). First, all samples were serially diluted (1 : 100, 1 : 1000, 1 : 10 000) and analyzed starting at the lowest dilution by NP100 (size range: 50–330 nm) and NP400 (185–1100 nm) nanopores stretched between 45 and 48 mm. Only measurements with a particle count > 500 or a time period of 5 min, linear particle rate in time, and noise below 15 pA were recorded. The pressure was adjusted to achieve a particle flow rate > 100/s and a stable current between 120 and 150 nA. Calibration was performed using calibration beads of a known concentration and size [CPC100 (110 nm) and CPC400 (340 nm), both from Izon Science] diluted at 1 : 1000 according to the manufacturer’s protocol. All samples, including calibration samples, were vortexed for 30 s before obtaining measurements. Data were analyzed by control suite V3.3 software for qNano (Izon Science).

### Transmission electron microscopy

Samples were stored at −80 °C prior to analysis. EV preparations were thawed on ice, and 25 µL from each sample was negatively stained. Prior to fixation, liver‐ and heart‐derived EVs were diluted at 1 : 100 in filtered 1× PBS. EVs were fixed with 2.5% glutaraldehyde in phosphate buffer [1 : 1 (v:v) ratio] overnight for more than 18 h. The sample (5 µL) was loaded on collodion‐coated 150‐mesh copper grids (#651; Nisshin EM Co., Ltd., Tokyo, Japan) and air‐dried for 40–60 min. Grids were then washed in 1 mL of ultrapure water (3 × 5 min), air‐dried (20–30 min), and stained with 15 µL of 3% phosphotungstic acid (#162‐02432; FUJIFILM Wako; dissolved in 1× PBS and filtered through a 0.22‐μm membrane) for 5 min. Immediately after staining, the excessive staining solution was blotted, and the sample was washed in 1 mL of ultrapure water (3 × 5 min) and air‐dried for 20–30 min. Four grids per each sample were prepared, and images were obtained by a transmission electron microscope (JEM‐1011; JEOL, Tokyo, Japan) at 80 kV.

## Results

While there is currently no consensus on definite specific markers for each EV subtype [21], characterization of the EVs collected in this study followed the criteria recommended by the International Society of EVs [21]. That is, small EVs were vesicles smaller than 150 nm and large EVs were vesicles larger than 150 nm.

### Liver‐derived EVs

Previous studies targeting tissue‐derived EVs involved short‐term culture of tissues explants or extraction from whole tissues [6, 7, 11, 12, 13, 15, 22, 23]. Tissue processing is particularly crucial for tissue extraction of EVs because mechanical disruption may release the intracellular content, leading to contamination. Jang *et al*. and Vella *et al*. [13, 19] reported collecting exosomes from mechanically disrupted tissues. We used both intact and cut liver tissues in *ex vivo* tissue culture and compared the efficiency of the methods using a precipitation kit and ultracentrifugation.

**Fig. 2:**
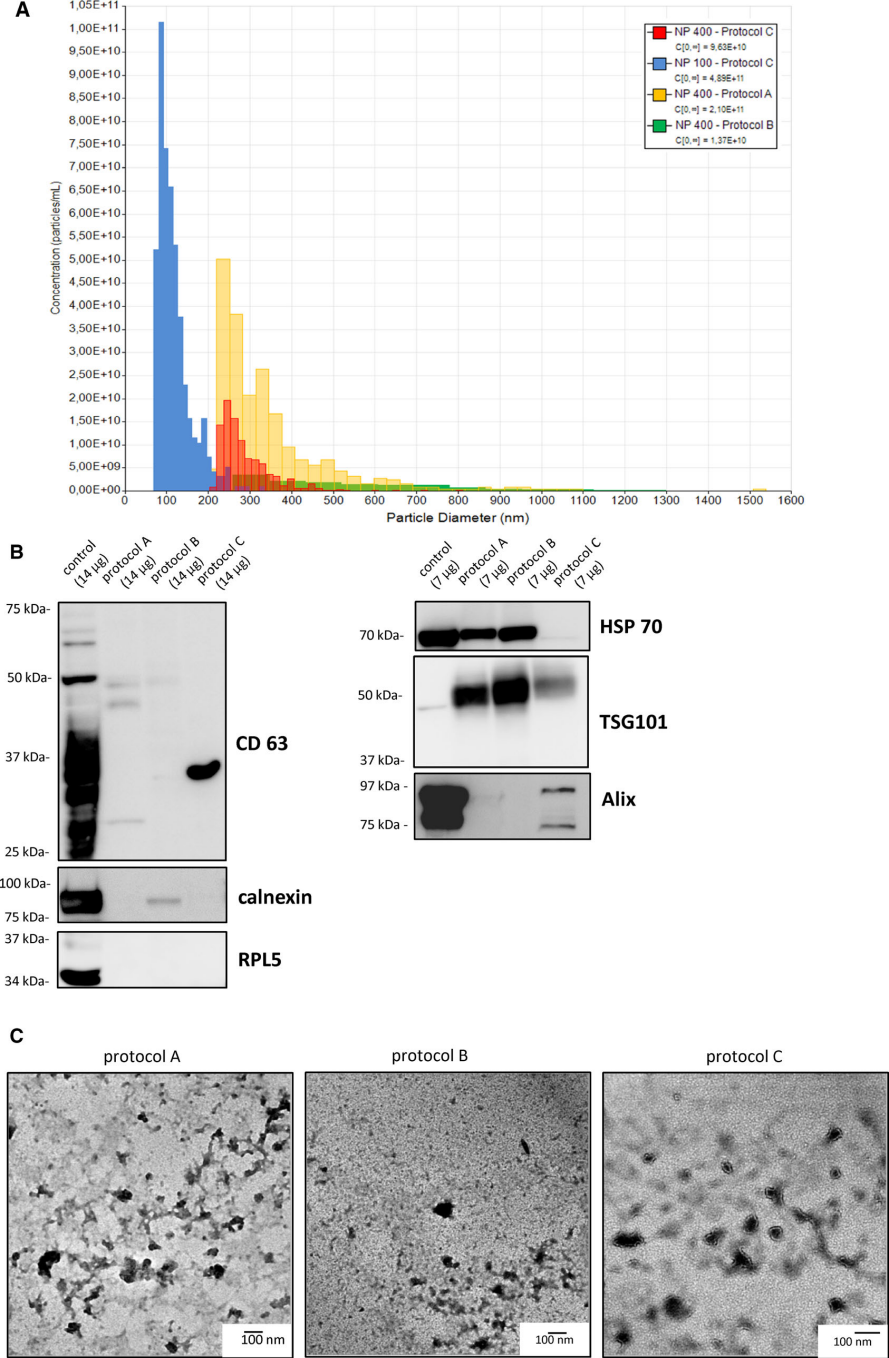
Characterization of EVs collected from damaged liver (comparison of three protocols). (A) Particle size distribution of collected samples as measured by TRPS by NP100 (small EVs) and NP400 (large EVs) nanopore. The analysis revealed the presence of both large (> 150 nm) and small (< 150 nm) EVs. Particle diameter (nm) in Protocol C (NP 100): mean (SD) 117 (40.6), mode 86; Protocol C (NP 400): mean 287 (65.9), mode 245; Protocol A (NP 400): mean 346 (152.9), mode 236; and Protocol B (NP 400): mean 540 (232.7), mode 303. Samples from Protocols A and B could not be analyzed by NP 100 nanopore due to constant blockage of the nanopore. (B) Western blot for HSP70, Alix, CD63, TSG101, RPL5, and calnexin. The total amount of protein loaded was 14 μg per sample for CD63, RPL5, and 7 μg for HSP70, Alix, TSG101, and calnexin. Corresponding whole‐cell lysate as a positive control was loaded—HEK293T for CD63 and HeLa for all other markers. (C) EV samples collected by the three protocols were negatively stained and observed by TEM. Scale bar: 100 nm.

To evaluate the size distribution of collected vesicles, freshly collected samples from the liver were subjected to TRPS analysis (Fig. 2A). We used an NP100 nanopore membrane (pore size diameter: 50–330 nm) for the small EVs and NP400 (185–1100 nm) for the large EV measurements. Samples from Protocols A and B caused constant nanopore blocking, unstable current, and noise exceeding 15 pA, even at higher dilutions and thus could not be analyzed by NP100 membrane. Only Protocol C samples (Fig. 2A; blue color graph) could be analyzed using the NP100 membrane (mean size: 117 ± 40.6 nm). In NP400 membrane analysis, we detected vesicles with the size > 150 nm in samples from all three protocols [Protocol A (yellow graph): 346 ± 152.9 nm, Protocol B (green graph): 540 ± 232.7 nm, Protocol C (red graph): 287 ± 65.9 nm]. The concentration of smaller EVs (4.89 × 10^11^ particles per mL) was higher than that of larger EVs (9.63 × 10^10^ particles per mL) in Protocol C.

We next analyzed several types of proteins associated with EVs. CD63, a tetraspanin protein highly expressed in EVs [24], was detected only in the sample from Protocol C (Fig. 2B). Regarding endosomal markers, Alix and TSG101, which are generally used to distinguish exosomes (small EVs) from other similar size vesicles for their role in exosomal biogenesis [24, 25], TSG101 was expressed in the samples obtained from all the three protocols, whereas Alix was predominantly detected only in the Protocol C sample. HSP70, a commonly detected protein in EVs [25], was recognized in the samples from Protocols A and B, but to a lesser extent in the sample from Protocol C (Fig. 2B).

Samples were also examined for proteins thought to be relatively depleted in EVs versus cells. Their detection would indicate contamination with intracellular components that are unlikely to be packaged into EVs. Endoplasmic reticulum‐related calnexin was detected in Protocol B, while it was under the detection limit in Protocol A and Protocol C samples. The protein RPL5, a protein that comprises the 60S ribosomal subunit [13], was under the detection limit in all three samples.

In TEM, EV‐like vesicular structures were observed in the Protocol C sample, whereas the Protocol A and Protocol B samples mainly showed debris‐like structures and EV‐like structures were difficult to be detected (Fig. 2C).

Overall, the Protocol C sample seemed to contain a higher amount of smaller size EVs (mean vesicle size: 117 ± 40.6 nm) with an EV‐like morphology. Those EVs were positive for EV‐associated markers, CD63, Alix, TSG101, and HSP70, with a lesser extent of contamination on the basis of the calnexin and RPL5 expression. On the other hand, Protocols A and B yielded vesicles contaminated with non‐EV content and some of the EV‐associated markers were under detection limit.

### Skeletal muscle‐derived EVs

**Fig. 3:**
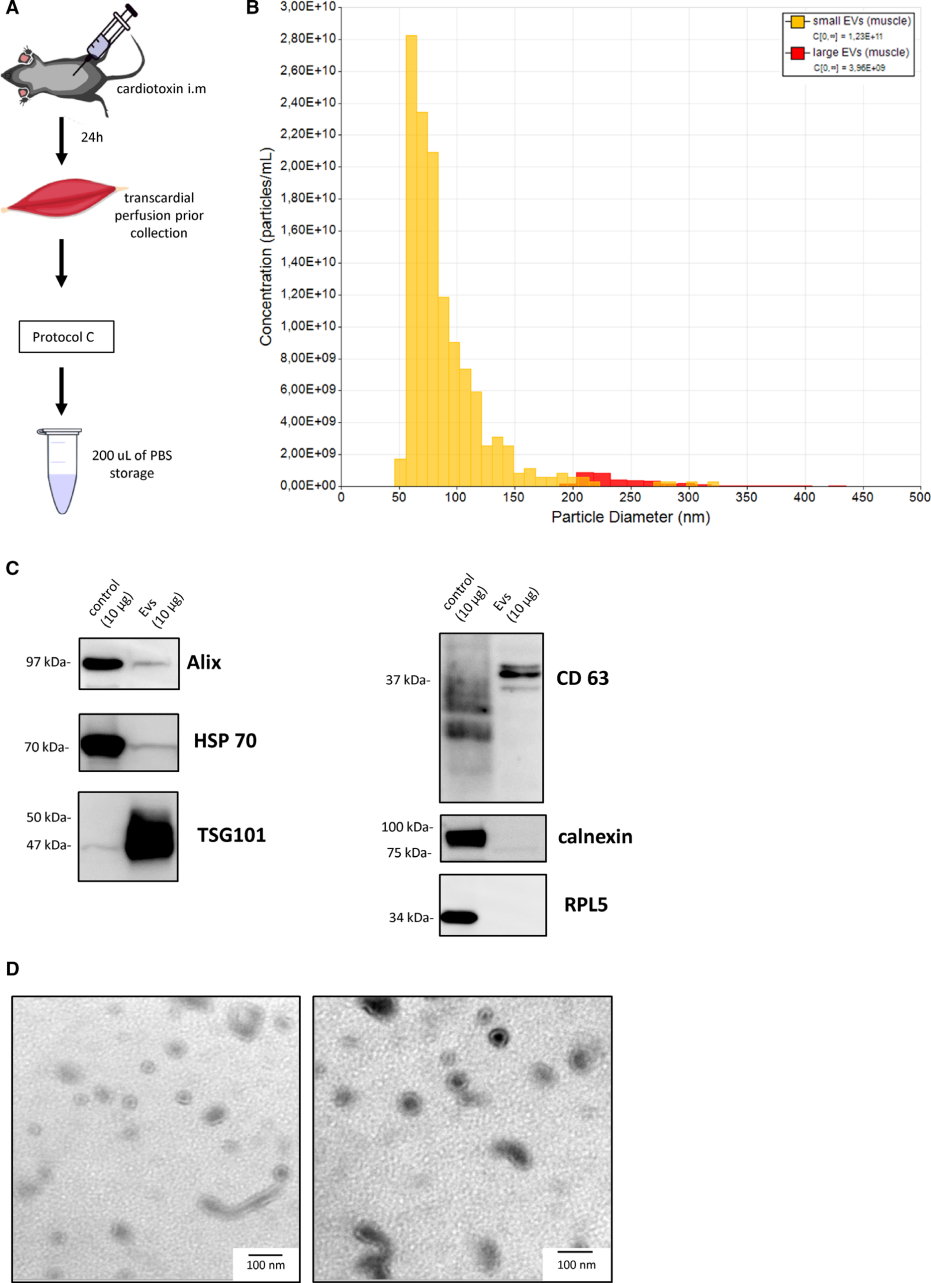
Characterization of EVs collected from damaged skeletal muscle. (A) Schematic diagram of the EV separation protocol from the damaged skeletal muscle. (B) Concentration and size diameter of collected EVs as measured by TRPS. Small EVs: mean (SD) 88 (35.5), mode 60; and large EVs: mean 260 (80.8), mode 210. (C) Western blot for Alix, HSP 70, TSG101, CD63, calnexin, and RPL5 (10 μg of total protein loaded, control—corresponding whole‐cell lysate as a positive control was loaded—HEK293T for CD63 and HeLa for all other markers). (D) Representative TEM of EVs derived from skeletal muscle. Scale bar: 100 nm.

Based on the above result, we applied Protocol C to damaged skeletal muscle tissue (Fig. 3A). Similar to the liver, a heterogeneous mixture of large and small EVs was collected. TRPS analysis showed a proportion of large EVs (260 ± 80.8 nm) among the small EVs (88 ± 35.5 nm) with a concentration of 3.96 × 10^9^ and 1.23 × 10^11^ particles per mL, respectively (Fig. 3B). Western blot detected expression of TSG101 and CD63 in the EV preparation (Fig. 3C). These results suggest less contamination because the calnexin and RPL5 levels were under the detection limit (Fig. 3C). Similarly to Protocol C in the liver sample, TEM showed the presence of EV‐like vesicular structures with sizes of ~ 100 nm (Fig. 3D).

### Heart‐derived EVs

**Fig. 4:**
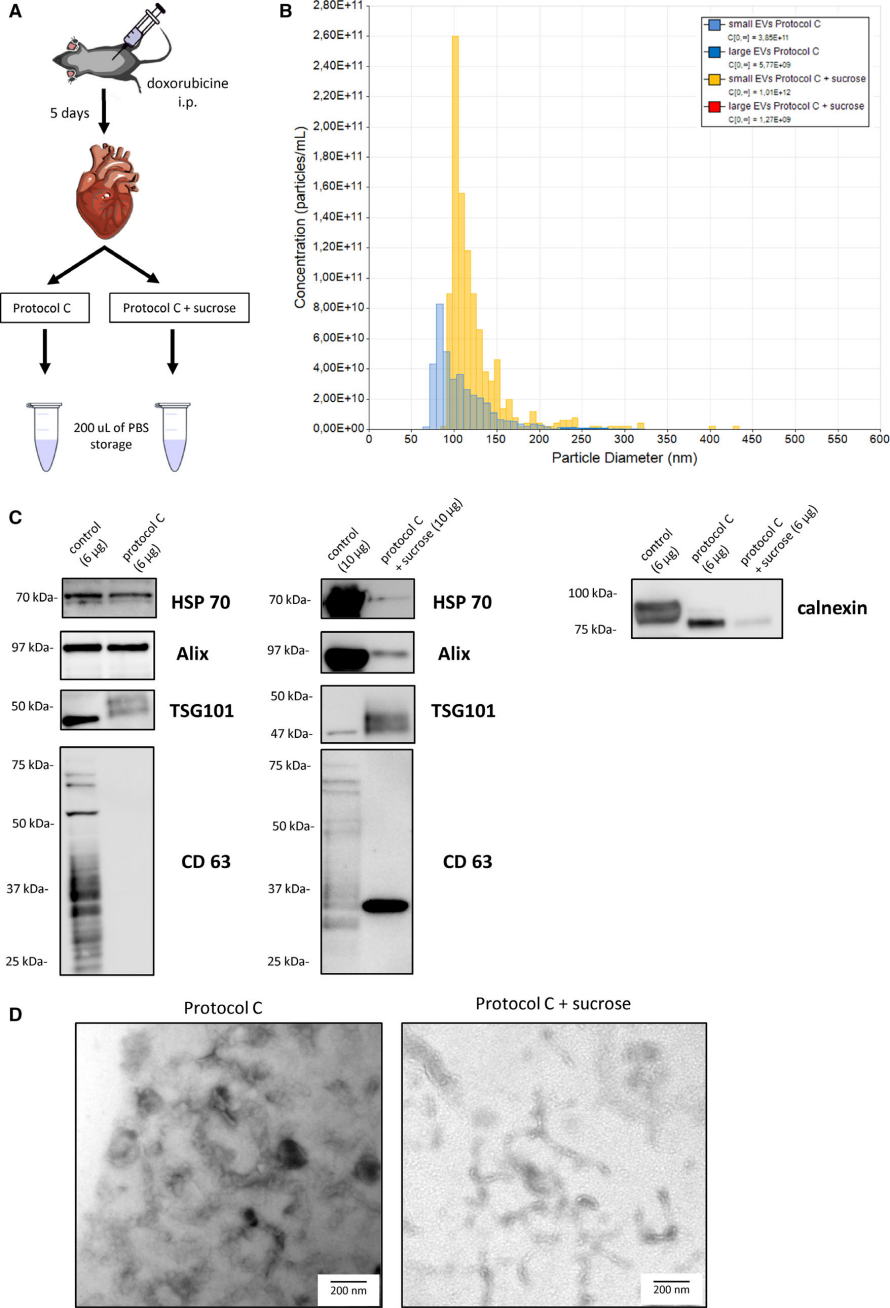
Characterization of EVs collected from damaged heart tissue by Protocol C and Protocol C + sucrose. (A) Schematic of the EV separation protocol from heart tissue. (B) TRPS analysis of the EV size distribution. Small EVs (Protocol C + sucrose): mean (SD) 125 (39.8), mode 102. Large EVs (Protocol C + sucrose): mean 398 (167.5), mode 281. Small EVs (Protocol C): mean 108 (33.5), mode 83. Large EVs (Protocol C): mean 302 (112.9), mode 238 (C) Western blot for HSP70, Alix, TSG 101, CD63, and calnexin. The total amount of protein loaded was 10 μg (Protocol C + sucrose) or 6 μg (Protocol C). For calnexin, an equal amount of total protein from both collection methods was loaded (6 μg). Corresponding whole‐cell lysate as a positive control was loaded—HEK293T for CD63 and HeLa for all other markers. (D) Representative TEM picture of EVs derived from damaged heart tissues collected by Protocol C and Protocol C + sucrose. Scale bar: 200 nm.

We first applied Protocol C to damaged hearts, although we could not detect CD63 in the EV preparation (Fig 4C). We then applied a simple modification of Protocol C by adding a sucrose cushion step (Fig. 4A) to determine whether CD63 is expressed in heart‐derived EVs [7, 20]. Heart‐derived EV preparations collected by these protocols (Protocol C and the combination of Protocol C with sucrose cushion, called ‘Protocol C + sucrose’ in the following sentences) showed a size range of < 150 nm for small EVs (exosomes) with a mean vesicle size of 108 ± 33.5 and 125 ± 39.8 nm by Protocol C and Protocol C + sucrose, respectively (Fig. 4B). The concentration of small EVs was higher in the sample collected by Protocol C + sucrose compared with that collected by Protocol C (1.01 × 10^12^ and 3.85 × 10^11^ particles per mL, respectively). The size and concentration of large EVs collected by Protocol C and Protocol C + sucrose were 398 ± 167.5 nm with 1.27 × 10^9^ particles per mL, and 302 ± 112.9 nm with 5.77 × 10^9^ particles per mL, respectively. Total protein yield was higher in the EV preparation collected by Protocol C + sucrose; therefore, a different amount of maximum total protein was loaded for western blot in the samples acquired by both methods. HSP70, Alix, and TSG101 were detected in the samples from both methods except CD63, which was only detected in Protocol C + sucrose (Fig. 4C). Equal amounts of protein from both methods were analyzed by western blot for calnexin, which was expressed at higher levels in the sample collected by Protocol C than in that collected by Protocol C + sucrose (Fig. 4C). EV‐like vesicular structures were observed in both preparations (Fig. 4D).

## Discussion

In the present study, we collected EVs from solid tissues—the liver, muscle, and heart. Collected tissue‐derived EVs exhibited characteristics similar to those of EVs from cell culture or biofluids, in accordance with the minimal information for studies of extracellular vesicles (MISEV2018) recommendations by the International Society of EVs [21]. The tissue was either cultured *ex vivo* as a tissue explant or enzymatically and mechanistically disrupted prior to the centrifugation steps.

The precipitation method is an easy‐to‐use approach for collecting EVs, particularly in culture medium and some biofluids [9, 26]. When applied to the liver, however, Protocols A and B yielded large EVs and non‐EV contaminants, as suggested by constant nanopore blockage by TRPS, which was not observed in the sample from Protocol C. EV aggregates are a main cause of nanopore blockage that can be prevented by thorough vortexing before the measurement. We carefully vortexed the samples before every measurement in TRPS. These aggregates would usually be detected as vesicles with a size 2–3 times bigger than that of small EVs. We detected vesicles > 1 µm in size in both EV preparations using the precipitation kit in Protocols A and B. Additionally, precipitation kits are reported to co‐isolate contaminating factors or nonexosomal impurities from the cell culture medium [27]. The expression of CD63 and Alix proteins was mainly observed in Protocol C, and not in Protocols A and B. Similarly, as in some other tissue‐derived EVs, we observed EV‐like structures by TEM in our preparations. Together, these findings suggested that EV preparations obtained by ultracentrifugation contain vesicles more comparable to endosome‐derived exosomes with less contamination of intracellular components, compared with the ready‐to‐use precipitation kit. Similar results were observed by Van Deun *et al*. [27] in EVs from cell culture medium, when comparing a precipitation kit and ultracentrifugation method.

Interestingly, adding a sucrose cushion as a modification of Protocol C for the heart improved the overall yield of EVs, vesicle count, total EV protein yield, contamination, and CD63 expression (CD63 was not detected by basic ultracentrifugation). All of these parameters differ among cell types in cell culture or biofluids [28] and might also be applicable to tissue‐derived EVs. It may also depend on the tissue type or collection method, as was demonstrated by the lower detection of calnexin in heart tissue after adding the sucrose cushion. Depending on the downstream application and overall grade of EV purity, including an additional step such as a density gradient [13, 19] may be beneficial.

Several protocols for tissue‐derived EVs are available, targeting a specific single type of tissue [6, 7, 11, 12, 13, 15, 19]. Our data show that a tissue‐specific approach may be necessary and more suitable than a universal collection method. The starting weight of the tissue may significantly contribute to the overall yield of EVs and associated proteins. Perfect separation of EV subpopulations remains technically difficult, and precise specific markers for EVs are not yet defined at the cell culture level [21].

Current studies of EVs are mostly based on those collected from cell culture or biofluids [9]. While the method described here targeted the liver, skeletal muscle, and heart, it can likely be applied to other tissue types as well. We realize that modifications of *ex vivo* culture medium to preserve the natural condition and integrity of solid tissue or applying sucrose cushion in all tissues for comparison could affect the results. This was not part of the scope of the present study, but should be investigated in the future. EV content characterization such as the RNA profile or proteomics would also be beneficial and requires further investigation.

## Conflict of interest

The authors declare no conflict of interest.

## Author contributions

All the authors planned experiments and analyzed the data. AM performed the experiments; and AM and MD wrote the manuscript.

## Data Availability

Data are available from the corresponding authors upon reasonable request.
